# Toward the appropriate interpretation of Alphafold2

**DOI:** 10.3389/frai.2023.1149748

**Published:** 2023-08-15

**Authors:** Tian Xu, Qin Xu, Jianyong Li

**Affiliations:** ^1^Department of Biochemistry, Virginia Polytechnic Institute and State University, Blacksburg, VA, United States; ^2^Department of Mathematics, The University of Arizona, Tucson, AZ, United States

**Keywords:** deep learning, machine learning, Alphafold2, protein, protein folding

## Abstract

In life science, protein is an essential building block for life forms and a crucial catalyst for metabolic reactions in organisms. The structures of protein depend on an infinity of amino acid residues' complex combinations determined by gene expression. Predicting protein folding structures has been a tedious problem in the past seven decades but, due to robust development of artificial intelligence, astonishing progress has been made. Alphafold2, whose key component is Evoformer, is a typical and successful example of such progress. This article attempts to not only isolate and dissect every detail of Evoformer, but also raise some ideas for potential improvement.

## 1. Introduction

The most common approach to obtain and elucidate protein structures in biological science is x-ray crystallography, by which three-dimensional protein structures are derived based on x-ray diffraction data of individual amino acid residues (Smyth and Martin, 2000). Another useful tool to derive protein structure is nuclear magnetic resonance (NMR) spectroscopy (Wüthrich, 1990), by which one can obtain dynamic changes of the protein structures under specific conditions. Both approaches often lead to accurate structural solutions of proteins of interest. In addition, protein NMR analysis could provide dynamic changes of a given protein under specific solvent conditions. However, obtaining structural information is extremely time consuming and expensive with either method. Issues related to obtaining protein samples with adequate purity also present themselves in protein crystallography and NMR analysis. For example, obtaining crystals that diffract at high resolution once protein samples are obtained is a challenge in protein crystallography. In protein NMR analysis, the size and solubility of given protein samples may lead to additional problems and may require more sophisticated NMR equipment. Some membrane proteins are difficult to crystallize, if not impossible.

Despite the painstaking nature of structural determination (by crystallography or NMR), structural determination is essential, providing structural information for a deep understanding of individual protein functions and numerous reliable models for comparison and contrast. The availability of a substantial number of protein structures is essential, particularly through the crystallographic approach. This requirement is based on the need for a solid foundation in understanding protein functions and interactions at a molecular level. Achieving these structures through crystallography demands a high degree of accuracy during the crystallization process. A known downside of NMR spectroscopy is the huge number of purified samples needed. Managing the purifying process for large amounts of protein is challenging in some scenarios. There is a long history of protein folding prediction via computational methods. In 1995, the Critical Assessment of protein Structure Prediction (CASP) was founded. Held every 2 years, CASP provides an opportunity for scientists and engineers to test their mathematical models. However, despite decades of work, people have still not found a model to predict protein folding that is close to the ground truth.

In 2020, Alphafold2 (Jumper et al., 2020) won the CASP14 for achieving the most accurate prediction of protein-folding with less than the diameter of one carbon atom confidence error in average (< 1Å in average). This was the best result made for that time, and it vastly outperformed other competitors' results. The competitor with the highest scores obtained a summed z score of 244.0217 and an average Z score of 2.6524. In comparison, the closest competitor achieved a lower summed z score of 92.1241 and an average Z score of 1.0013.^1^ This result was exhilarating since it was the first-time humans had begun getting truly close to the ground truth. Moreover, scientists have started applying Alphafold2 in many sub-fields of biology. For example, Ren et al. applied Alphafold2 in drug discovery. As the first reported small molecule targeting CDK20, out of the seven compounds that were synthesized, ISM042-2-001 exhibited a Kd value of 9.2 ± 0.5 μM (*n* = 3) in the CDK20 kinase binding assay (Ren et al., 2022). In the work by Zhang et al. (2020, 2021), they even extended Alphafold2 by combining Prod Conn. AlphaFold2, and sequential Monte Carlo (Doucet et al., 2001) to predict engineered unstable protein structures. Their findings indicate that the representations derived from AlphaFold2 models can effectively forecast the stability alterations caused by point mutations. Furthermore, they observed that AlphaFold accurately predicted the ProDCoNN-designed sequences, which exhibited varying root-mean-square deviations (RMSDs) in relation to the target structures. This suggests that certain sequences possess higher foldability compared to others (Zhang et al., 2021). There are more examples in Table 1. Since the major components of Evoformer (Jumper et al., 2020) (row and column attention, triangular self-attention) (Vaswani et al., 2017) are all built upon the transformer model (Vaswani et al., 2017), it is important to have a good grasp of the transformer model (Vaswani et al., 2017). In this article, we will highlight the structure of the Evoformer (Jumper et al., 2020) and how it works from a basic attention mechanism (Vaswani et al., 2017), for instance, the math behind it and what the attention score really is. We will also discuss how Evoformer relates to transformer (Vaswani et al., 2017) and the difference between them, especially when message passing based on graph theory (Gilmer et al., 2020) is blended in. Despite its strengths, Alphafold2 shows reduced accuracy when predicting very long sequences. The root mean square deviation (RMSD) of longer sequence prediction grows quickly. A potential improvement for this weakness is provided in the discussion section.

**Table 1:** Applications of Alphafold2 in different areas.

	**Area of study**	**Achievement**
Novel fold of rotavirus glycan-binding domain predicted by AlphaFold2 and determined by X-ray crystallography (Hu L. et al., 2022)	Structural biology	Alphafold2 successfully predicts the structure of the VP8^*^ domain (VP8^*^B) of VP4 and the result is verified by experiment.
AlphaFold accelerates artificial intelligence powered drug discovery: efficient discovery of a novel Cyclin-Dependent Kinase 20 (CDK20) small molecule inhibitor (Ren et al., 2022)	Drug discovery	Alphafold2 successfully predicts the structure of the chemical compound which demonstrates it is helpful in the early stage of drug discovery.
De novo protein design by inversion of the AlphaFold structure prediction network (Goverde et al., 2023)	Protein design	Alphafold2 shows the potential capability to solve the challenge in de novo protein design.
CavitySpace: a database of potential ligand binding sites in the human proteome (Wang et al., 2022)	Target prediction	Alphafold2 is used to create the database CavitySpace which is the first library of human proteome.
Exploring evolution-based and-free protein language models as protein function predictors (Hu M. et al., 2022)	Protein function prediction	Evolution-based evoformer and evolution-free evoformer are compared based on alphafold2.
Improved prediction of protein-protein interactions using AlphaFold2 (Bryant et al., 2022)	Protein-protein interaction	Alphafolder2 based docking methods perform better than other docking methods.
Identification of a novel substrate motif of yeast separase and deciphering the recognition specificity using AlphaFold2 and molecular dynamics simulation (Liang et al., 2022)	Biological mechanism of action	Alphafold2 helps scientists with deeper understanding of mechanism of substrate recognition and activation of separase.

## 2. Methodology

Many machine learning techniques have been used in Alphafold2, including Evoformer (Jumper et al., 2020) and dataset distillation (Wang et al., 2018). In this article, we will mainly focus on the Evoformer, a modification of the transformer (Vaswani et al., 2017), because Evoformer is the most crucial component of Alphafold2.

Evoformer is a derivative of the transformer (Vaswani et al., 2017), and it improves attention modules to fit many proteins' specific aspects. In the transformer model (Vaswani et al., 2017) for Image Recognition at Scale (ViT) (Dosovitskiy et al., 2020) or Natural Language Processing (NLP), embedding vectors are not correlated to each other. However, from an Evoformer perspective, all the rows and columns are somehow interconnected. So, to solve the interconnections in that scenario, Evoformer introduced row-wise and column-wise attention, which have practical meanings in homology.

One of the inputs of Evoformer is Multiple Sequence Alignment (MSA) (Corpet, 1988), which is composed of human amino acids sequences and amino acid sequences from other animals that are highly similar or identical to humans. Row attention tracks the amino acids' correlations among different species and column attention compares the interconnections among amino acids sequences among homologous species.

Another important input of Evoformer is amino acids pairs, which allow protein-to-protein information transfer (Root-Bernstein, 1982). One of the major problems of sequential data machine learning in a Euclidean space is it does not sustain the relational connectivity for neighborhood elements, especially when we assume relational connectivity does not have connection, but it does. The consequence of this is the model will lose tons of information to induce the result. To maintain the neighborhood, Evoformer aggregates information across amino acids pairs by using Triangular multiplicative updates and Triangular self-attention in analogy to passing information in GNN (Gilmer et al., 2020). In this article, we will mainly focus on the structure analysis of Evoformer and the comparison between the traditional transformer (Vaswani et al., 2017) and Evoformer.

### 2.1. Linear projection

In linear algebra, a linear projection P is a linear function to a vector space that, when applied to itself, elicits the same result. This definition of linear projection is confusing because it is too abstract to imagine. In machine learning, linear projection is used to project a target vector onto higher or lower dimensional subspace and simply align different vectors' dimensions. Perceptron (Marsalli, 2008) is a typical linear projection usage.

In transformer models (Vaswani et al., 2017), including Evoformer, embedding vectors are projected into query, key, and value vectors. Assume input $X={\mathbb{R}}^{m\times n\;},W_{K},W_{Q}={\mathbb{R}}^{n\times d_{k}}\;and\;W_{V}={\mathbb{R}}^{n\times d_{l}}$


(1) $$\begin{array}{l} Q=X\cdot W_{Q} \end{array}$$

(2) $$\begin{array}{l} K=X\cdot W_{K} \end{array}$$

(3) $$\begin{array}{l} V=X\cdot W_{V} \end{array}$$

where $Q,K\in {\mathbb{R}}^{m\times d_{k}}$ and $V\in {\mathbb{R}}^{m\times d_{l}}$. *Q, K*, and *V* stand for query, key and value. *W*_*Q*_, *W*_*K*_, *and W*_*V*_ are learnable parameters. The intuition of why input *X* is projected to three different outputs is from a retrieval system. A query is like your search input in Google; Google finds your expected value based on the key words of your search.

### 2.2. Attention


(4) $$Attention\left(Q,K,V\right)=softmax\left(\frac{Q\cdot K}{\sqrt{d_{k}}}\right)\cdot V$$

**Figure 1:**
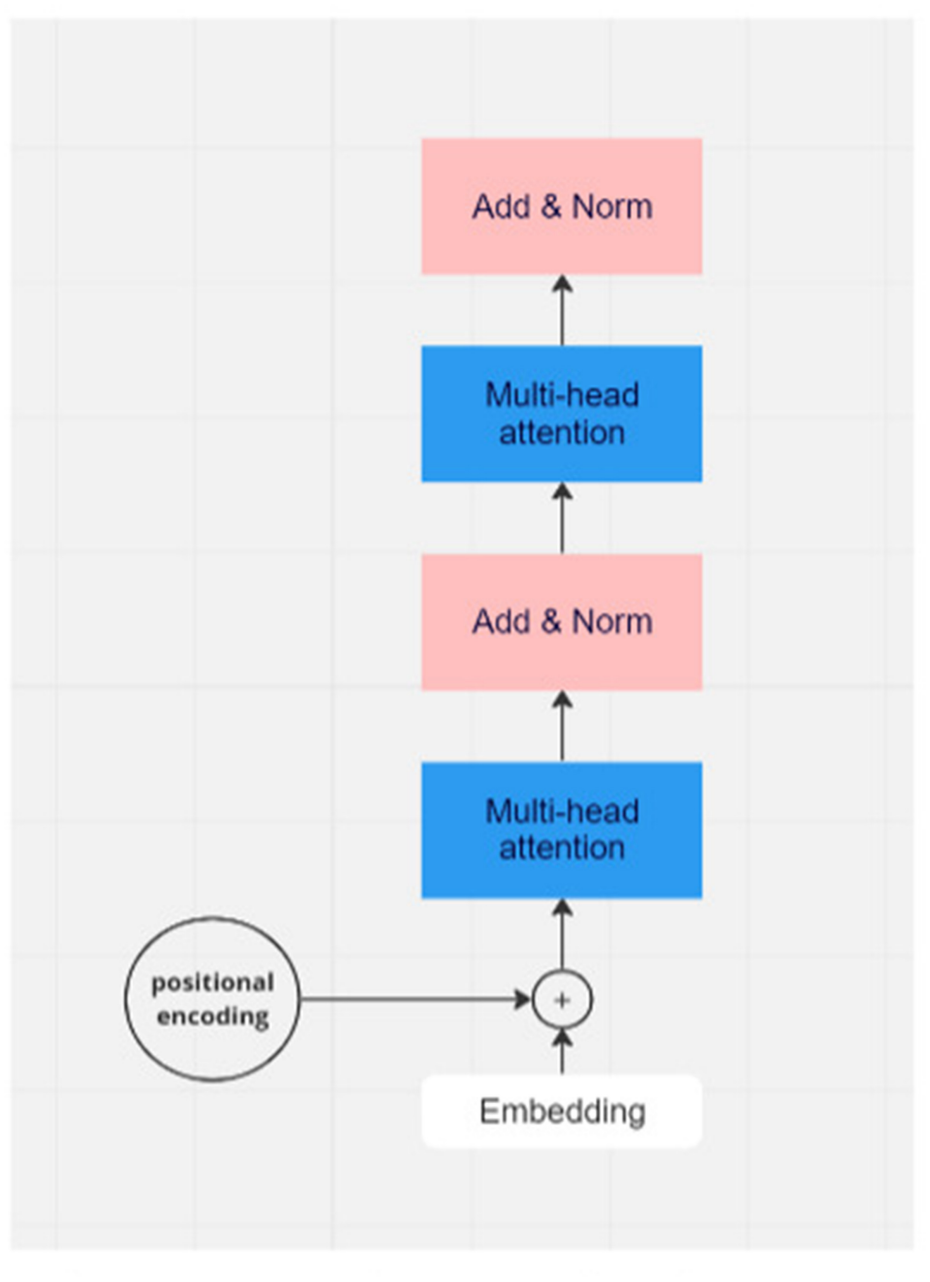
Flowchart of one module of transformer model. In real applications, there would be many layers of transformer modules.

Attention mechanisms can be analogous to humans capturing crucial information in sentences. The transformer model (Vaswani et al., 2017) utilizes dot-product attention instead of additive attention for efficient storage and faster performance (Figure 1). To understand why a dot product works in attention, we need to look inside the dot product.

Let's assume two vectors **u** and **v** in ℝ^*n*^:


(5) $$u\cdot v=||u||\cdot ||v||\cdot \cos\theta_{uv}=\;\sum_{i=1}^{n}u_{i}\cdot v_{i}$$

The Euclidean distance between vectors **u** and **v**:


(6) $$\begin{array}{l} ||u-v||\;=\;\sqrt{||u|{|}^{2}+\;||v|{|}^{2}-2\cdot ||u||\cdot ||v||\cdot \cos\theta_{uv}}\\ \;=\sqrt{||u|{|}^{2}+\;||v|{|}^{2}-2\cdot u\cdot v} \end{array}$$

**Figure 2:**
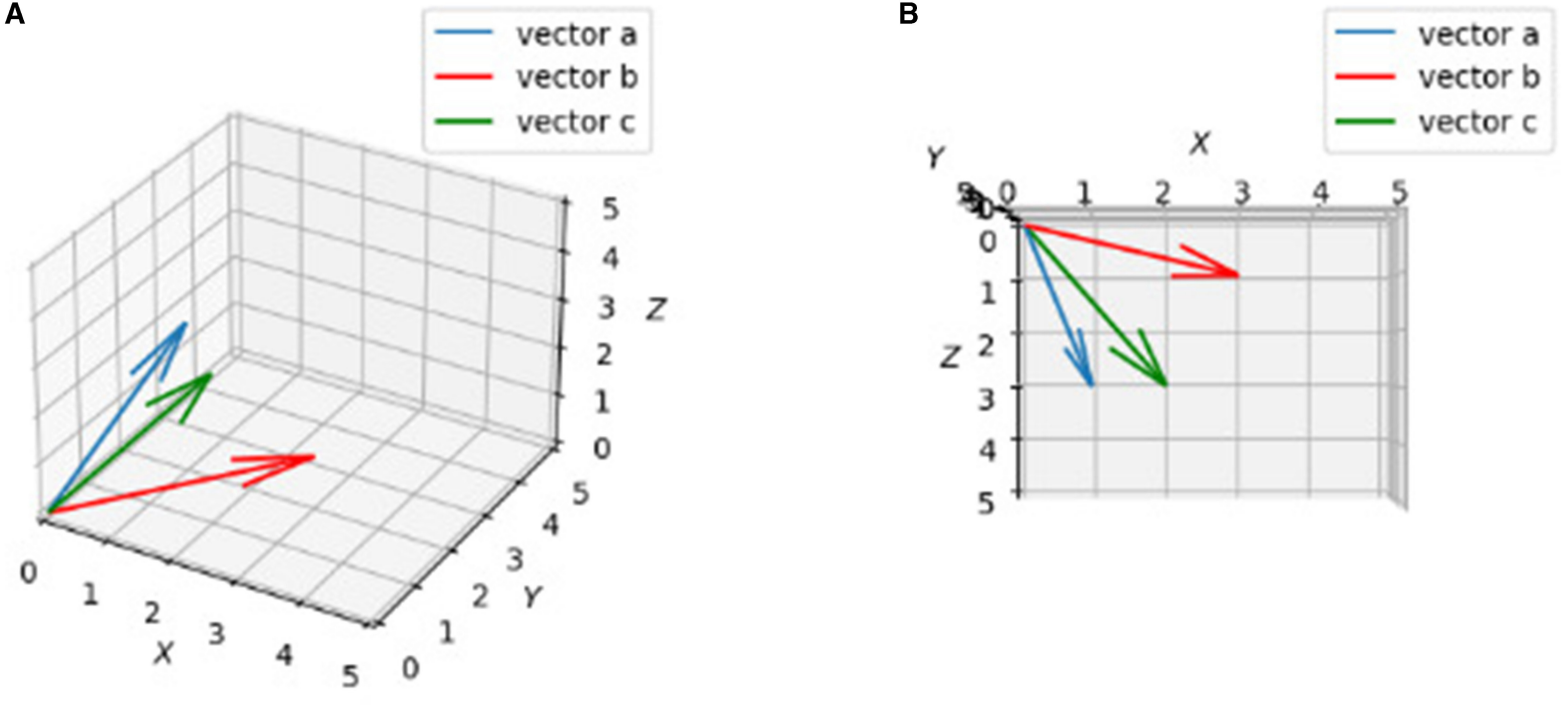
Plots of three vactors a, b, c. 3D drawings of vectors a = [1, 2, 3], b = [3, 2, 1], c = [2, 1, 3]. **(A)** Rough view in 3D space. It may not show c is “closer” to a than b obviously. **(B)** Projection in X-Z plane. By looking from top to bottom (projection), it is clearly to say c is “closer” to a than b.

In general, if two vectors are close, their Euclidean distance needs to be small according to equation 6, which means the value from equation 5 needs to be big enough. In this manner, one can say the bigger the dot product value, the more similar the two vectors are. Although we cannot draw diagrams in dimensions higher than three, we can show what vectors in 3d looks like in terms of similarity (Figure 2):


$$\begin{array}{l} a=\left[\begin{array}{ccc} 1 & \&\;2 & \&\;3 \end{array}\right],\;b=\left[\begin{array}{ccc} 3 & \&\;2\;\& & 1 \end{array}\right],\;c=\left[\begin{array}{ccc} 2 & \&\;1\;\& & 3 \end{array}\right]\\ \;||a||=\;||b||=\;||c||=\;\sqrt{14}\\ \;a^{T}\cdot b=1\cdot 3+2\cdot 2+3\cdot 1=10\\ \;a^{T}\cdot \;c=\;1\cdot 2+2\cdot 1+3\cdot 3=13\\ \;||a-b||=\;\sqrt{28-2\cdot 10}=\;\sqrt{8}\\ \;||a-c||=\;\sqrt{28-2\cdot 13}=\;\sqrt{2}\\ \;||a-b||>\;||a-c|| \end{array}$$

This is exactly how self-attention works. The scalar value calculated from the dot product of two vectors is represented as scores of similarities. In equation 4, *Q* is a matrix and *K* is also a matrix. To calculate dot products of each row between matrix *Q* and *K*, matrix multiplication is required:


$$\begin{array}{l} Q\cdot K^{T}=q_{1}\cdot k_{1}+q_{2}\cdot k_{2}+\cdots +q_{i}\cdot k_{i}=\overset{m}{\underset{i=1}{\sum }}q_{i}\cdot k_{i} \end{array}$$

The problem of dot-product attention is it may get either too large or too small depending on the *d*_*k*_. To see that, assume all *q*_*i*_ and *k*_*i*_ are random independent variables and have a distribution with mean 0 and variance 1


(7) $$\begin{array}{l} Cov\left(q_{i},k_{i}\right)=E[(q_{i}-E\left[q_{i}\right])(k_{i}-E[k_{i}])]\\ \;=E\left[q_{i}\cdot k_{i}\right]-E\left[q_{i}\right]\cdot E[k_{i}]=0 \end{array}$$

(8) $$\begin{array}{l} Var\left(q_{i}\right)=E\left[q_{i}^{2}\right]-E{\left[q_{i}\right]}^{2}\\ \;=E[q_{i}^{2}]\\ \;=1 \end{array}$$

(9) $$\begin{array}{l} Var\left(k_{i}\right)=1 \end{array}$$

So, based on equations 8 and 9, we have:


(10) $$\begin{array}{l} Var\left(q_{i}k_{i}\right)=E{\left[(q_{i}k_{i}\right)}^{2}]-E{\left[q_{i}k_{i}\right]}^{2}\\ \;=E{\left[q_{i}\right]}^{2}\cdot E{\left[k_{i}\right]}^{2}-{\left(E\left[q_{i}\right]\cdot E\left[k_{i}\right]\right)}^{2}=1 \end{array}$$

and *k*_*i*_ is a random independent variable and based on equation 7, 10:


(11) $$E\left[q\cdot k\right]=\;\sum_{i=1}^{d_{k}}E\left[q_{i}\cdot k_{i}\right]=0$$

(12) $$Var\left(q\cdot k\right)=\;\sum_{i=1}^{d_{k}}Var\left(q_{i}\cdot k_{i}\right)=d_{k}$$

(13) $$Softmax\left(x_{i}\right)=\;\frac{e^{x_{i}}}{\sum_{j}e^{x_{j}}}$$

If *d*_*k*_ is large enough, **qk** may be much larger or smaller than mean 0.

**Figure 3:**
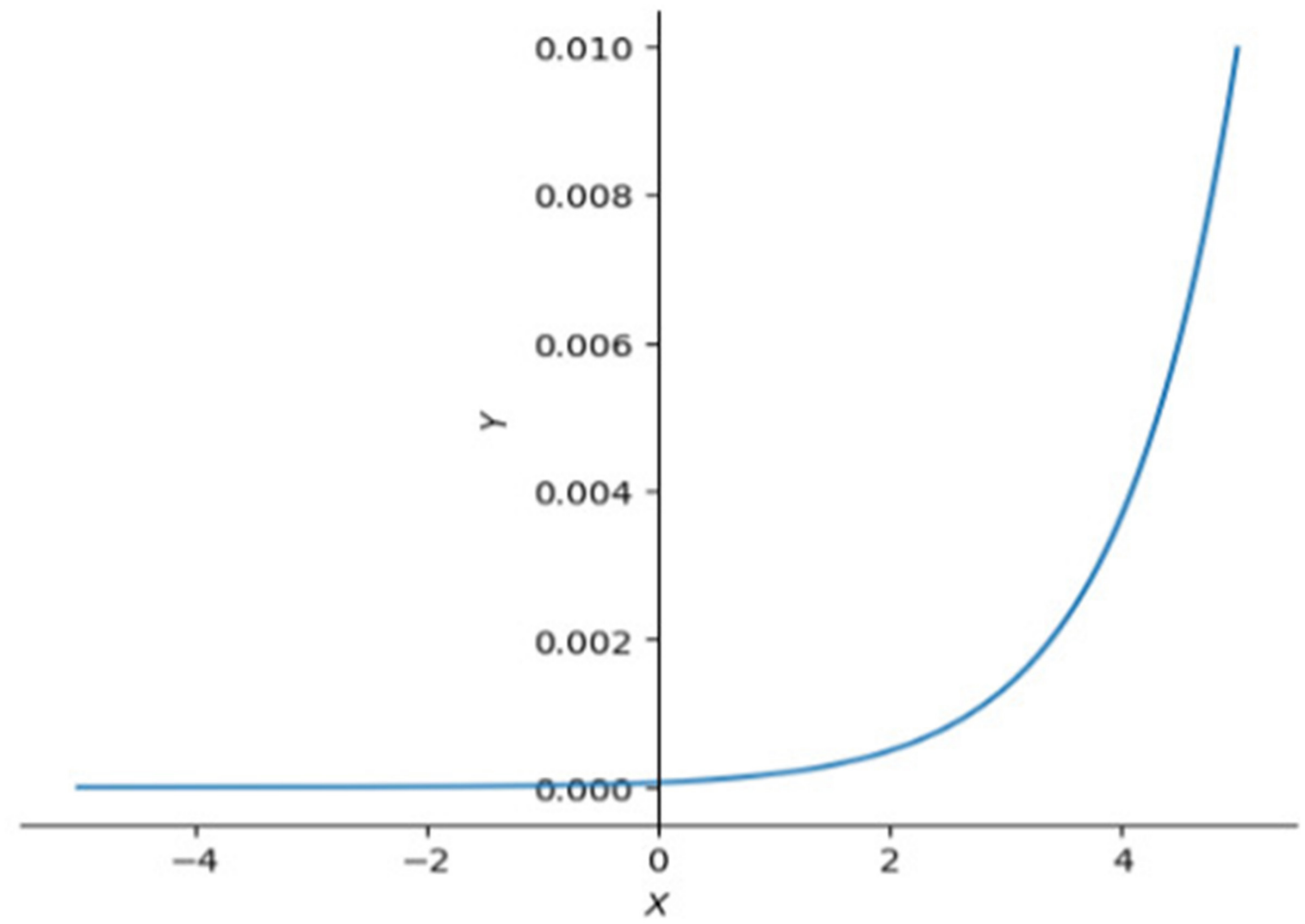
SoftMax functions in 2D. This graph illustrates that the right side of SoftMax function is close to horizontal line which means the slope in that interval is also close to 0, left side of SoftMax function goes up dramatically making the slope approach infinity. Large slope value is also not expected sometimes, especially when model is reaching optimal.

This will make the SoftMax (equation 13) activation function in equation 4 have an unstable or tiny slope (Figure 3), and make the gradient at the backpropagation stage explode or vanish. To avoid that happening, we need to clip it by scaling down the scores by their standard deviation ($\sqrt{d_{k}}$) (equation 12).


(14) $$\begin{array}{l} a_{i}=Softmax\left(\frac{q_{i}\cdot k_{i}}{\sqrt{d_{k}}}\right) \end{array}$$

(15) $$\begin{array}{l} attention\left(q_{i},k_{i},v_{i}\right)=a_{i}\cdot v_{i} \end{array}$$

### 2.3. Multi-head

**Figure 4:**
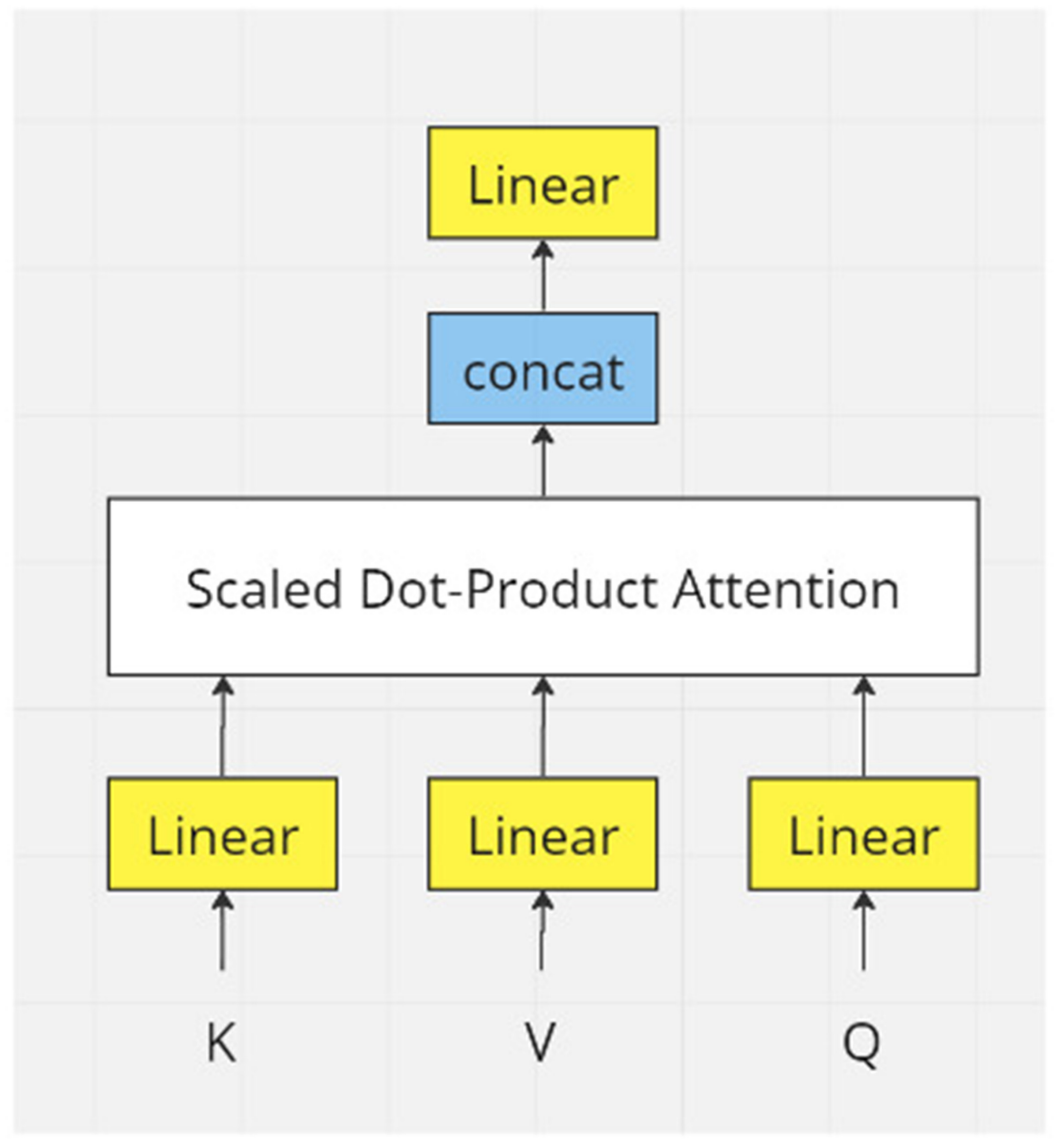
Workflow of one multi-head attention unit.

In the transformer model (Vaswani et al., 2017), linear projection and attention have been done h (h = 8 in transformer model (Vaswani et al., 2017) times (Figure 4). So, equations 1, 2, and 3 become:


(16) $$\begin{array}{l} \;Q_{i}=X\cdot W_{Q_{i}}\\ \;K_{i}=X\cdot W_{K}{}_{i}\\ \;V_{i}=X\cdot W_{V_{i}}\\ i=1,2,3,\cdots ,h \end{array}$$

equation 4 becomes:


(17) $$\begin{array}{l} Head_{i}=Attention\left(Q_{i},K_{i},V_{i}\right)=Softmax\left(\frac{Q_{i}\cdot K_{i}}{\sqrt{d_{k}}}\right)\cdot V_{i}\\ \;i=1,2,3,\cdots ,h \end{array}$$

To get all attention, we concatenate all the heads by:


(18) $$\begin{array}{l} Heads=Concat\left(Head_{1},Head_{2},\cdots \;,Head_{h}\right) \end{array}$$

Recalling equations 1, 2, and 3, the input dimension is ℝ^*m*×*n*^. The result of equation 18 has the dimension ℝ^*m*×*nh*^. To keep all attention layers consistent, we use a matrix $W_{o}={\mathbb{R}}^{nh\times n}$ to learn a representation back to dimension ℝ^*m*×*n*^.'


(19) $$\begin{array}{l} MultiheadAttention\left(Q,K,V\right)=Heads\cdot W_{o} \end{array}$$

### 2.4. Residual

Theoretically, a deeper network should learn more representations than a shallow network. In practice, this is not true since gradients may vanish in deep layers. Intuitively, we want deep layers to perform at least as well as shallow layers, not worse. Resnet (He et al., 2016) introduced a way to solve that problem called “Identity shortcut connection.” If a model is already optimized, we have


(20) $$\begin{array}{l} F\left(x\right):=H\left(x\right)-x=0 \end{array}$$

Equation 20 means *H*(*x*) is an identical mapping of x and optimal layers learn nothing. So, it makes sure that the shallow layers receive useful information(*x*) at least.

### 2.5. Layer normalization


(21) $$\begin{array}{l} x_{i}=\;\frac{x_{i}-\;\overset{\bar{}}{x}}{\delta } \end{array}$$

(22) $$\begin{array}{l} \underset{n\to \infty }{\lim}\overset{n}{\underset{i=1}{\sum }}\frac{x_{i}}{n}=\mu \;\left(law\;of\;large\;numbers\right) \end{array}$$

**Figure 5:**
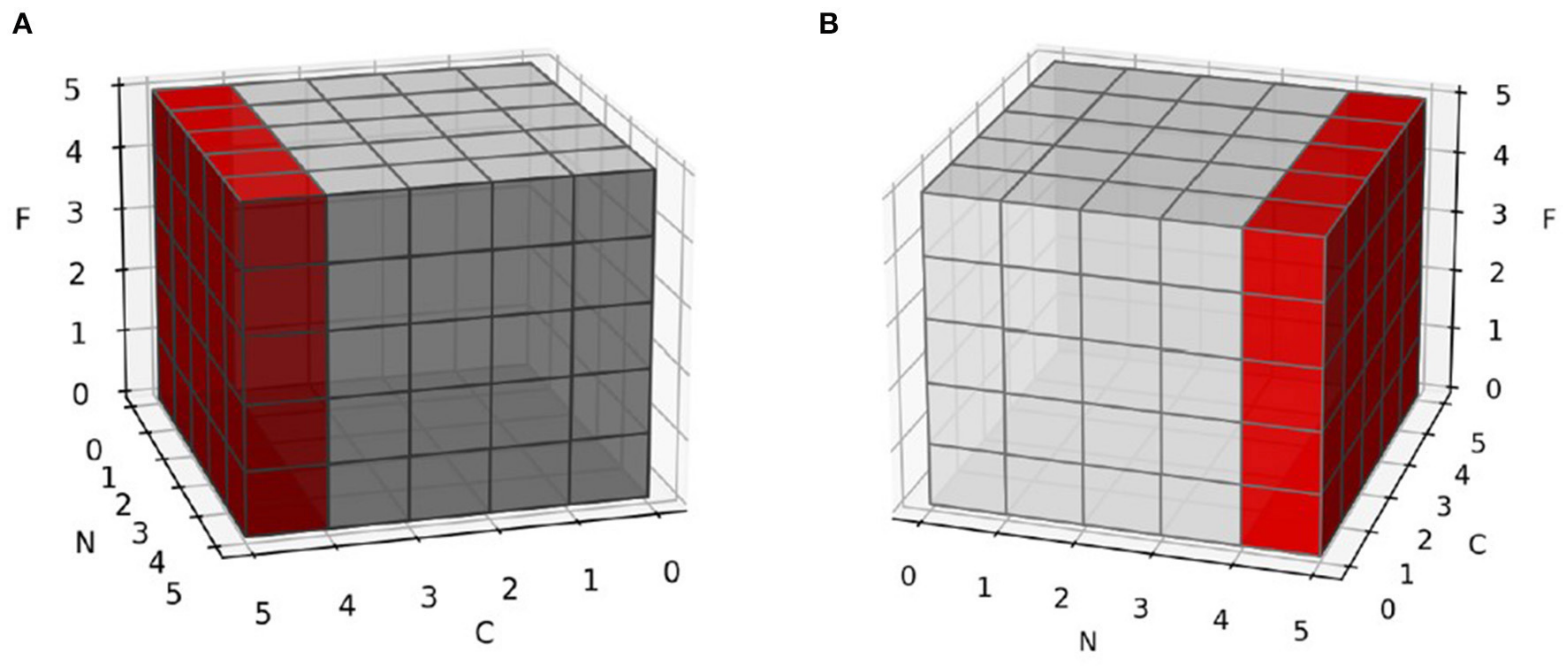
Normalization in different axis. C is channel or embedding axis, N is batch axis and F is feature axis. **(A)** Normalize samples in batch axis. Each feature is not normalized in sample-wise, but in batch-wise. **(B)** Normalize samples in their channel or embedding axis. Each feature is not normalized in batch-wise, but in sample-wise.

Layer normalization (Ba et al., 2016) is a substituent of batch normalization (Ioffe, 2015). It provides great help in two areas of batch normalization: small batch sizes of samples and different input lengths in dynamic networks. Both areas mean batch normalization cannot represent the real distribution of data (equation 21 and 22). Instead of normalizing across the batch axis, layer normalization normalizes the channel or embedding axis to avoid such problems (Figure 5).

## 3. Evoformer

With previous knowledge of the transformer model (Vaswani et al., 2017), we can continue with Evoformer, which takes some advantages from the transformer model, but also provides improvement to fit specific protein attributes. In this section, we give a brief introduction of how Evoformer works and is related to the transformer model.

### 3.1. Row and column attention

Like equation 1, the transformer model (Vaswani et al., 2017) is often used in NLP missions. The batched inputs of NLP are word-token sequences where they are not related at all if random sampling is performed. For instance, the first sentence is “Alphafold is a great tool,” and the next sentence is “Panda is one of the rare animals,” but that is not the case in Evoformer. The “word-tokens” from input layers are homologous to each other among rows and columns. Row-wise sequence alignment could represent the similarity or difference of gene expression across different species and column-wise sequence alignment embeds the information of how gene expression evolves (Zhang et al., 2022). For such reasons, Evoformer is used for how to learn weight distribution across rows and columns. Supplementary Figure 2 from the original paper (Jumper et al., 2020) shows all the steps of row-wise self-attention and looks like a traditional multi head self-attention in transformer model with several modifications. One can notice that queries (*r*_*q*_, *h, c*), keys(*r*_*v*_, *h, c*), values(*r*_*v*_, *h, c*), and gate(*r*_*q*_, *h, c*) are all linearly transformed from a specific specie (row-wise). To calculate attention weight, pair bias is added at the end before SoftMax activation is performed. Moreover, gate g is used to filter value representation.

For regular self-attention (equation 4) in the transformer model, there is no bias terms since it only has one source of input (e.g., sentences, pictures). However, Evoformer has two totally distinct types of input: MSA and amino acids pairs. Just like a linear function, for instance *y* = *x*·*w*+*b*, according to algorithm 7 from Supplementary material of the paper (Jumper et al., 2020), if we treat $\frac{q_{si}^{h^{T}}\cdot k_{sj}^{h}}{\sqrt{c}}$ as *xw* and $b_{ij}^{h}$ as *b*, then $b_{ij}^{h}$ shifts the attention score to fit the actual score better by utilizing the information in pairs. The sub-index of each tensor is a little bit tricky. So be cautious when aligning the proper $b_{ij}^{h}$ to $\frac{q_{si}^{h^{T}}\cdot k_{sj}^{h}}{\sqrt{c}}$.

As mentioned above, column-wise attention is a new technique for specific protein problems. It captures attention across MSA columns. Columns in MSA represent the same gene expression among different species. Supplementary Figure 3 (Jumper et al., 2020) from the original paper shows the details of column-wise attention. Queries (*s*_*q*_, *h, c*), keys(*s*_*v*_, *h, c*), values(*s*_*v*_, *h, c*), and gate(*s*_*q*_, *h, c*) are all linearly transformed from a specific gene expression (column-wise). There is no pair bias anymore so that column-wise attention looks almost the same as the transformer's self-attention except it has a gate.

**Figure 6:**
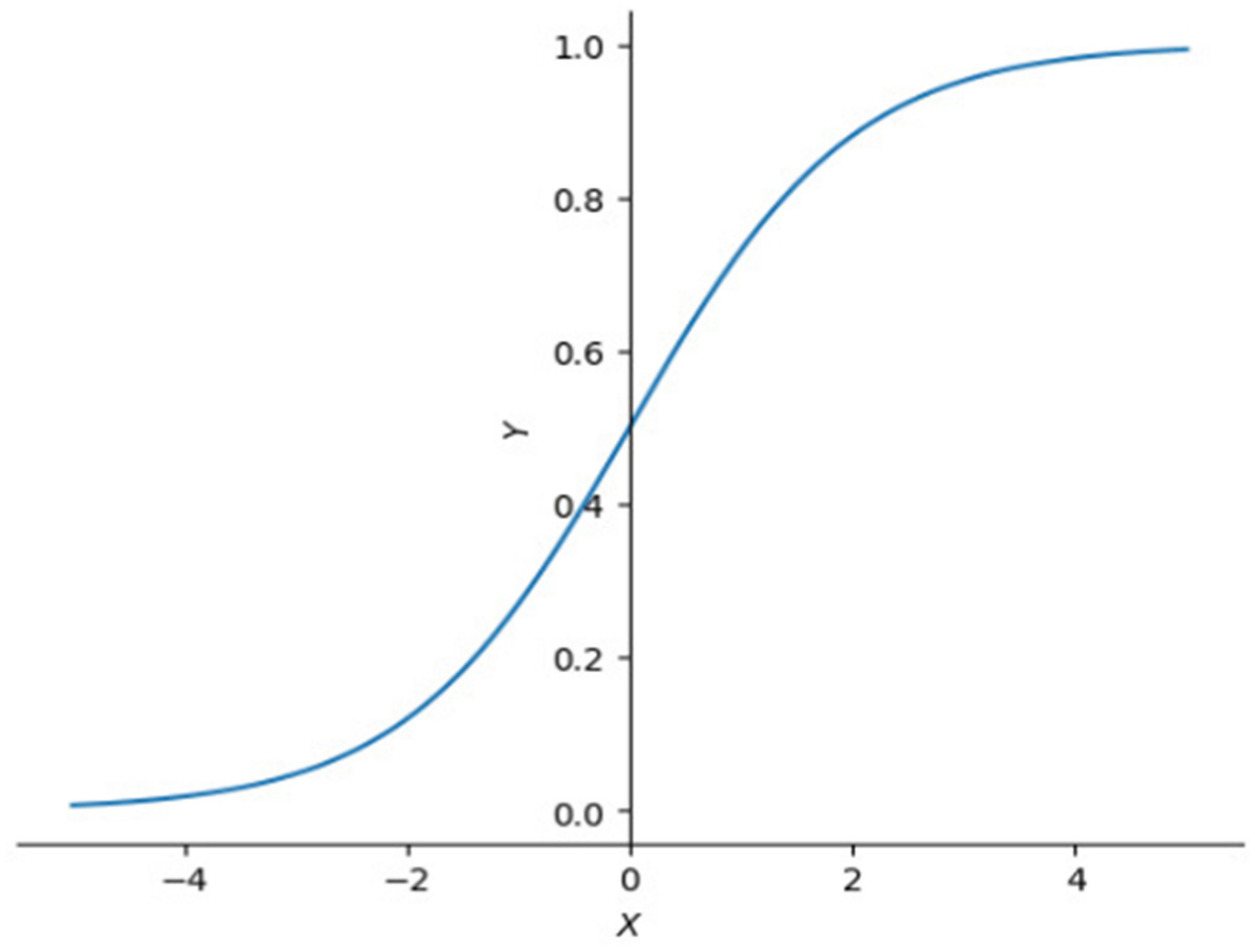
Plot of sigmoid function. Sigmoid function is often used as filter because it compresses input values between 0 and 1. Close to 1 if input value is large enough or 0 if input value is small.

The idea of a gate has a relatively long history. Long short-term memory (LSTM) (Hochreiter, 1997) introduced a few kinds of gate cell to improve RNN (Medsker, 2001). A gate cell can be seen as a filter which only allows values with high confidence to pass because of the nature of sigmoid function (Figure 6).

### 3.2. MSA transition

MSA transition is equivalent to a feed forward layer in the transformer model (Vaswani et al., 2017) which has most of the trainable parameters and key-value memories (Geva et al., 2020).

### 3.3. Outer product mean

As in Figure 5A of the paper (Jumper et al., 2020), the result coming from the transition layer needs to be added back to pair representation. However, MSA has a shape (*s, r, c*_*m*_) and pairing representation has a shape (*r, r, c*_*z*_). To add them up, MSA must be reshaped to the same shape as the pairing representation [Supplementary Figure 5 of the paper (Jumper et al., 2020)]. The outer product is a vector version of the Kronecker product of matrices. The most straightforward effect of the outer product is that the outer product of two vectors is a matrix. In other words, an extra dimension is produced. Here is an example:

Assume vectors **u** and **v** ∈ ℝ^*n*^


(23) $$\begin{array}{l} u\;⊗v=\;\left[\begin{array}{c} u_{1}\\ u_{2}\\ u_{3}\\ \vdots \\ u_{n} \end{array}\right]\;⊗\;\left[\begin{array}{ccccc} v_{1} & v_{2} & v_{3} & \cdots & v_{n} \end{array}\right]\\ =\left[\begin{array}{ccccc} u_{1}v_{1} & u_{1}v_{2} & u_{1}v_{3} & \cdots & u_{1}v_{n}\\ u_{2}v_{1} & u_{2}v_{2} & u_{2}v_{3} & \cdots & u_{2}v_{n}\\ u_{3}v_{1} & u_{3}v_{2} & u_{3}v_{3} & \cdots & u_{3}v_{n}\\ \vdots & \vdots & \vdots & \ddots & \vdots \\ u_{n}v_{1} & u_{n}v_{2} & u_{n}v_{3} & \cdots & u_{n}v_{n} \end{array}\right] \end{array}$$

To update pair representation *p*_*ij*_, it calculates the outer product of *i*th and *j*th columns from MSA representation, then takes the mean value along the new axis (first dimension *s*) and finally maps to pair representation *p*_*ij*_ with a linear transformation.

There are multiple ways to expand dimensions in this case. For example, we can perform a matrix tile operation on the third dimension. The reason to choose the outer product here is it condenses all the information in MSA in order to reconstruct pair representation.

### 3.4. Triangular multiplicative update

The triangular multiplicative update model is analogous to nodes in graph theory. To update the information contained at position {*i, j*} by receiving all information, its neighbor nodes {*i, k*} and {*j, k*} pass according to Supplementary Figure 6 of the paper (Jumper et al., 2020). There are two symmetric versions for updating *z*_*ij*_ (Jumper et al., 2020). One refers to the “outgoing” edge (row wise) and another is the “incoming” edge (column wise). In the “outgoing” edge version, every *z*_*ij*_ is updated by the sum of all columns of the *i*th row and *j*th row. In the “incoming” edge version, every *z*_*ij*_ gets updated by the sum of all rows of the *i*th column and *j*th column. The algorithm looks like row- or column-wise attention, but the dot product is replaced by elementwise multiplication for a cheaper computational cost (equation 24).

### 3.5. Triangular self-attention

Triangular self-attention looks almost exactly the same as row and column attention [algorithm 11 and 12 from the paper (Jumper et al., 2020)] and there are also two symmetric versions (Jumper et al., 2020). In the starting node version, the key **k**_*ij*_ and value **v**_*ij*_ are replaced by **k**_*ik*_ and **v**_*ik*_ (key and value from *i*th row and *k*th column), *b*_*jk*_ is also created and added to inner product affinities as a bias term. In the ending node version, the key **k**_*ij*_ and value **v**_*ij*_ are replaced by **k**_*ki*_ and **v**_*ki*_ (key and value from *i*th column and *k*th row); *b*_*ki*_ is also created and added to inner product affinities as a bias term. Now, updating **z**_*ij*_ not only depends on the dot product similarities of neighbor gene expression but also depends on the third edge *b*_*ik*_ and *b*_*kj*_ (Jumper et al., 2020).

## 4. Discussion

Although Alphafold2 can make highly accurate predictions of protein structures, it is not perfect. There are some things the model ignores.

(a) Alphafold2 is not designed to model co-factor-based protein folds. Myoglobin or hemoglobin need a heme to fold and zinc-finger domains are not stable without a zinc ion, so Alphafill is developed to address such issues (Hekkelman et al., 2023).
(b) One major drawback of AF2, which has a significant impact on the field of biomedicine, is the inadequate quality of transmembrane protein models. Although the confidence indices for transmembrane segments can be reliable, the overall predicted topology of these models is incompatible with their insertion into a membrane bilayer (Tourlet et al., 2023).
(c) An inherent constraint of MSA (Multiple Sequence Alignments)-based methods like AlphaFold2 is their reliance on existing knowledge and datasets. While these methods can make educated estimates between known protein structures and potentially even make predictions around those known structures, they struggle to confidently and precisely predict entirely new configurations. Incorporating a molecular dynamics component is crucial to effectively model these novel configurations (Marcu et al., 2022).

Considering the above limitations, the model should aim to incorporate the effects generated by co-factors. To improve the prediction results of poor-quality transmembrane proteins, it should consider how to enhance the accuracy and reliability of their modeling. Additionally, the model should focus on generalizing to data not present in the existing databases, in order to improve and refine its predictions.

## 5. Conclusion

Alphafold2 is a complicated system. There are more than 60 pages in the supplementary document of Alphafold2 (Jumper et al., 2020). If we break it down to every step, hundreds of pages will be required. In this article, we only discussed the most important module-Evoformer. However, other modules also made huge contributions to its success, for instance, data preparation, IPA Module, and recycling. The argument is still controversial, but some discussion says Alphafold2 is the future of protein structure prediction (Al-Janabi, 2022). However, Alphafold2 provides us with a new way to study protein structure where we can combine experimental efforts with a powerful tool set. The Alphafold2 model can be used to generate more specific drugs and find more suitable animals to test medicines (Thornton et al., 2021), improve structural coverage of the human proteome (Porta-Pardo et al., 2022), and so on. Alphafold2 also shows its success in general sequential models other than NLP and image recognition tasks. At the beginning of the self-attention transformer model (Vaswani et al., 2017), most of the studies were based on NLP missions, for instance, “Bert” (Devlin et al., 2018). Now, the transformer model is also popular in biology.

## Author contributions

TX contributed to the design and conception of the study. QX contributed to all the details and mathematics of the study. JL contributed by editing and reviewing the manuscript. All authors contributed to the manuscript revision, read, and approved the submitted version.

## Footnotes

^1^ https://predictioncenter.org/casp14/zscores_final.cgi
